# Intestinal Parasitic Infection and Nutritional Status in Children under Five Years Old: A Systematic Review

**DOI:** 10.3390/tropicalmed7110371

**Published:** 2022-11-12

**Authors:** Nisa Fauziah, Jenifer Kiem Aviani, Yukan Niko Agrianfanny, Siti Nur Fatimah

**Affiliations:** 1 Division of Parasitology, Department of Basic Biomedical Science, Faculty of Medicine, Universitas Padjadjaran, Bandung 45363, Indonesia; 2 Laboratory of Parasitology, Faculty of Medicine, Universitas Padjadjaran, Bandung 45363, Indonesia; 3 Research Center for Care and Control of Infectious Disease, Universitas Padjadjaran, Bandung 45363, Indonesia; 4 Department of Biotechnology, School of Life Science and Technology, Institut Teknologi Bandung, Bandung 40132, Indonesia; 5 Division of Clinical Nutrition, Faculty of Medicine, Universitas Padjadjaran, Bandung 45363, Indonesia

**Keywords:** soil-transmitted helminth, water-borne parasitic infection, intestinal parasitic infection, *Ascaris lumbricoides*, Trichura trichiura, Giardia lamblia, hookworm, children under 5, stunting, wasting, underweight

## Abstract

Intestinal parasitic infections are common infectious diseases causing many health problems and impaired growth and physical development.. Children under five years old are the most vulnerable to infections, due to their immature immunity and feeding and exploratory behaviours. This systematic review aimed to assess the relationship between intestinal parasitic infections and undernutrition among children under 5 years old. Fifteen studies met the inclusion and exclusion criteria and were classified as high-quality studies. Twelve parasites were reported, including *Ascaris lumbricoides*, *Cryptosporodium* spp., *Entamoeba histolytica*, *Enterobius vermicularis*, *Giardia lamblia*, hookworm, *Hymenolepis nana*, *Strongyloides sterocalis*, *Taenia* spp. and *Trichuris trichuria*. Ascariasis is the most reported infection, with a prevalence ranging from 10.77% in Ethiopia to 57.14% in Malaysia, and is correlated with stunting (OR 2.17 (95% CI 1.14, 4.13), *p* = 0.02). Giardiasis is the second most reported infection, with a prevalence ranging from 4.43% in Ethiopia to 66.33% in the Central African Republic, and is related to an increased risk of stunting (OR 2.34 (95% CI 1.07, 5.10), *p* = 0.03)), wasting (OR 2.90 (95% CI 1.12, 7.49, *p* = 0.03)), and being underweight (OR 1.53 (95% CI 1.02, 2.29, *p* = 0.04)). The third and fourth most prevalent infections are *T. trichiura* and hookworm infections. Intestinal parasitic infections can occur very early in life and cause significant growth retardation. It is important to understand the prevalence and effects of infection based on the parasite species in order to implement therapeutic interventions and prevention controls.

## 1. Introduction

Intestinal parasitic infections are among the most common infectious diseases, affecting approximately 3.5 billion people every year and causing more than 450 million health problems, including diarrhoea, abdominal pain, undernutrition, general malaise and weakness, and impaired growth and physical development [1,2,3,4,5]. Over 267 million preschool-age and 568 million school-age children live in areas where soil-transmitted helminths, a major cause of intestinal parasitic infections, are endemic [6,7,8,9]. Foodborne parasitic infection is another way parasitic disease can be transmitted. According to the WHO, 23.2 million cases of foodborne parasitic disease and 45,927 deaths were reported up until 2010 (excluding enteric protozoa). An additional 67.2 million cases of illnesses were reported to be caused by foodborne enteric protozoa [10]. Severe infections resulted in severe disability and neurological and psychiatric diseases [10,11,12,13]. Children are at high risk for these infections and they often experience reinfection [14,15,16,17].

Undernutrition is a lack of energy and nutrient intake in a person’s diet. Anthropometric indicators are the only validated and approved method for measuring nutritional statuses. Wasting refers a person with a low weight-for-height value for their age. Stunting refers to a low height-for-age value, whereas being underweight refers to a low weight-for-age value. The World Health Organization (WHO) growth standard is often used to describe these criteria where children that exhibit signs of wasting, stunting, or being underweight report values more than two standard deviations below the population standard (z < −2) [18].

Based on its aetiology, undernutrition is divided into acute and chronic. Primary acute undernutrition in children is a result of insufficient food intake. Secondary acute undernutrition is typically caused by abnormal nutrient loss, increased energy expenditure, or decreased food intake. This condition frequently occurs in the context of underlying chronic diseases, such as chronic infection, chronic liver disease, cystic fibrosis, chronic renal failure, childhood malignancies, congenital heart disease, and neuromuscular diseases. The primary indicator of acute undernutrition is wasting [6]. Chronic undernutrition, on the other hand, is caused by a lack of specific nutrients at specific times during early childhood, which results in physical and cognitive growth inhibition. Stunting is the most commonly used indicator of chronic undernutrition [19,20,21,22].

Worldwide, the prevalence of undernutrition in children under five years old remains high. In 2020, 149.2 million stunted children and 45.5 million wasted children were reported, accounting for 22% and 6.7% prevalence, respectively. In 2020, three regions (West and Central Africa, East and Southern Africa, and South Asia), or 33 countries, had high stunting prevalence and at least 30% of children under five years old were affected. Meanwhile, more than half of all the wasted children lived in South Asia, with nearly a quarter in Sub-Saharan Africa. Indonesia has one of the highest rates of stunting and wasting worldwide, with a stunting rate of more than 30% and 10–15% prevalence in children under five [23].

Undernourishment increases the likelihood of infection and its severity, while also slowing recovery [24,25,26]. There is a relationship between undernutrition and the immune system, where undernutrition can lead to fatal disruptions in immune system development and vice versa. Immune system disruption increases a child’s vulnerability to parasitic infection. Repeated gut infections, on the other hand, may compromise epithelial integrity and reduce nutrition absorption. Thus, these two conditions form a vitiosus circulus [27,28,29].

Children under five years old are the most vulnerable to infections, due to their immature immune systems [30,31]. During early childhood, the immune system is still developing and is not yet fully developed. Passive IgG antibody transfer from breastfeeding, commonly performed until the child is two years old, provides critical early protection [30]. In toddlers to pre-schoolers, food neophobia and fussiness/picky eating, two food-related nondirective feeding behaviours, lead to a limited, unhealthy diet, affecting their weight and nutritional status [31]. Their developing sensory behaviours, such as touch perception and taste, cause them to put anything in their mouth, which increases the likelihood of contracting parasitic infections [32].

Children under five years old are said to be in their “golden years,” when their emotional, social, and cognitive development is at its peak [32]. As a result, this age group requires special attention. Two of the WHO’s long-term aims are to eliminate stunting in children under five and mortality/morbidity related to intestinal parasitic infections among preschool children by 2030 [33]. However, the correlation between intestinal parasitic infection and undernutrition in children under five years old has not been thoroughly studied. This systematic review aimed to assess the relationship between intestinal parasitic infections and undernutrition among children under 5 years based on prevailing evidence.

## 2. Materials and Methods

### 2.1. Literature Search and Identification

This systematic review was conducted according to the Preferred Reporting Items for Systematic Reviews and Meta-analyses (PRISMA) [34] reporting guidelines. PubMed/MEDLINE, Ovid, Embase, Scopus, Web of Science, and Google Scholar databases were used to collect publications up to 21 September 2021. The following search terms were applied: children AND pre-school age (nutritional status OR stunting OR wasting) AND (parasite OR helminth infection). Additional studies were identified through screening of the reference lists.

### 2.2. Inclusion and Exclusion Criteria

Studies were included if they focused on (1) children aged ≤ 5 years old, (2) the prevalence of stunting or wasting or underweight; (3) the status of intestinal parasitic infection during the period of study. Studies were excluded if (1) they did not include original data, such as reviews, systematic reviews, comments, or editorial letters, (2) they did not include a comparison group (children with a normal nutritional status), (3) they were not written in English, (4) they were unpublished papers, (5) they did not report the correlation between intestinal parasitic infection and nutritional status, (6) they reported the history of parasitic infection but not the condition during the period of study, (7) they used methods other than the WHO WAZ (Weight-for-Age Z-score), HAZ (Height-for-Age Z-score), or WHZ (Weight-for-Height Z-score) standards to define underweight, stunting, and wasting; (8) they reported comparisons as WAZ/HAZ/WHZ scores only and did not consider the prevalence of children that scored under -2 SD.

### 2.3. Data Collection and Analysis

The title and abstract of every article were independently reviewed by all authors. If the abstract met the inclusion criteria, the full text article was thoroughly reviewed. Reference lists for the identified publications were screened for previously unidentified but relevant studies. The following information was retrieved: author, year, country, type of study, inclusion and exclusion criteria, age of children at assessment, no. of cohorts and controls, reported intestinal parasite, outcome, outcome assessment method and confounding factors. The quality of the methodology was assessed using the Joanna Briggs Institute Critical Appraisal Tools Checklist for Analytical Cross-Sectional Studies [35].

### 2.4. Outcome Variables

The primary outcome reviewed in this article is prevalence of stunting, wasting, and underweight in children manifested with intestinal parasites compared to non-infected children. Stunting is defined as a height-for-age value that is lower than 2 standard deviations of the height-for-age WHO standard (<−2 SD HAZ). Wasting is defined as a weight-for-height value that is lower than 2 standard deviations of the weight-for-height WHO standard (<−2 SD WHZ). Underweight is defined as a weight-for-age value that is lower than 2 standard deviations of the weight-for-age WHO standard (<−2 SD WAZ). Prevalence of specific intestinal parasites manifestations per region of study andrisk factors that were reported to be significantly correlated with intestinal parasitic infection and/or undernutrition were also thoroughly reviewed.

### 2.5. Statistical Analysis

If more than one study reported any primary outcome for any of the parasite species, effect size analysis using the Mantel–Haenszel odds ratio at 95% confidence intervals was conducted. RevMan version 5.3 software (Cochrane Collaboration, London, UK) was used for these purposes. The inconsistency index (I^2^) test was performed to evaluate heterogeneity across the studies. The result is presented as a forest plot.

## 3. Results

**Figure 1:**
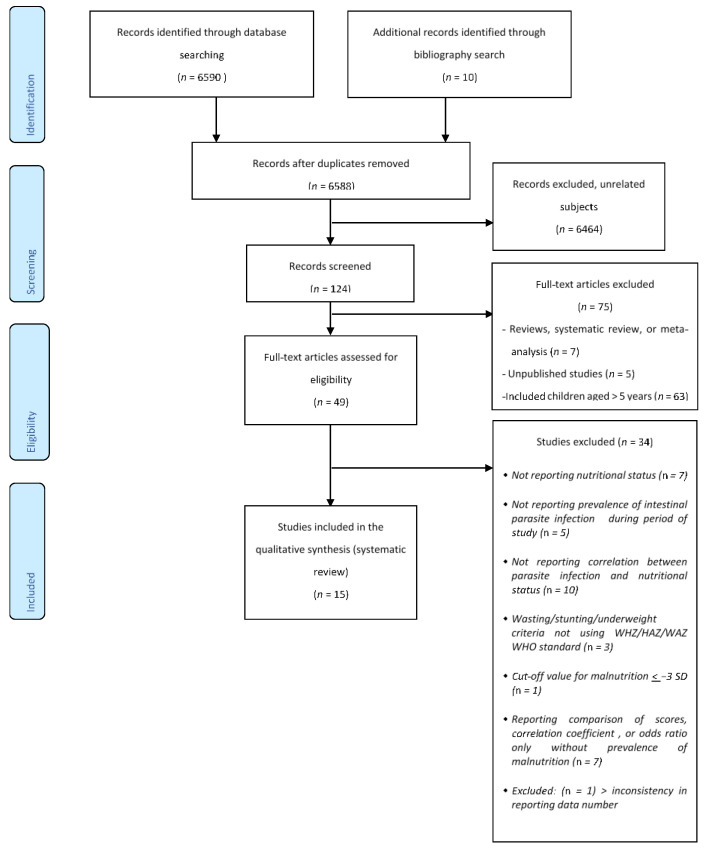
Study flow diagram.

The initial literature search yielded 6590 studies; 10 studies were identified through reference screening (Figure 1). Two articles were removed after being acknowledged as duplicates and after screening the titles and abstracts, 6464 articles were excluded, as they reported unrelated subjects. In total, 124 full-text articles were reviewed and only 49 of the articles fulfilled the inclusion criteria. The characteristics of the 49 full-text articles that were screened for eligibility are presented in Supplemental Table S1. Only 16 studies met the inclusion and exclusion criteria and were appraised [36,37,38,39,40,41,42,43,44,45,46,47,48,49,50,51]. The critical appraisal results for the included studies are presented in Supplemental Table S2 for cross sectional studies and Supplemental Table S3 for case-control study. One study was excluded due to inconsistency in the number of cohorts reported [51]. Description of the studies included in this review can be observed in Table 1.

### 3.1. Type of Parasite and Geographical Distribution

Twelve intestinal parasites were reported in the studies, including *Ascaris lumbricoides*, *Cryptosporidium* spp., *Entamoeba histolytica*, *Entamoeba vermicularis*, Giardia *lamblia*, hookworm, *Hymenolepis nana*, *Oxyuris* spp., *Strongyloides sterocalis*, *Taenia* spp. and *Trichuris trichuria*. *A. lumbricoides* infection was the most reported infection, which was stated in eight studies [36,37,41,43,45,47,48,49] and *G. lamblia* in eight studies [36,38,40,41,43,44,45,46], followed by *T. trichiura* in five studies [41,43,47,48,49], hookworm in four studies [41,45,47,49], *E. histolytica* in three studies [37,41,45], *S.sterocalis* in two studies [41,45], and the others only in one study, respectively. The studies were conducted in 12 countries and the prevalence of parasitic infections in these countries ranged from 35.08% to 51.78%, as can be observed in Table 2. *A. lumbricoides* infection was the most reported with the highest prevalence, ranging from 10.77% in Ethiopia to 57.14% in Malaysia. *G. lamblia* infection was the second most prevalent infection, with reported prevalence ranging from 4.43% in Ethiopia to 66.33% in the Central African Republic. The third and fourth most prevalent infections were *T. trichiura* and hookworm infection. As can be observed from the table, the prevalence of specific parasite infections was different according to the geographical region.

### 3.2. Stunting

**Figure 2:**
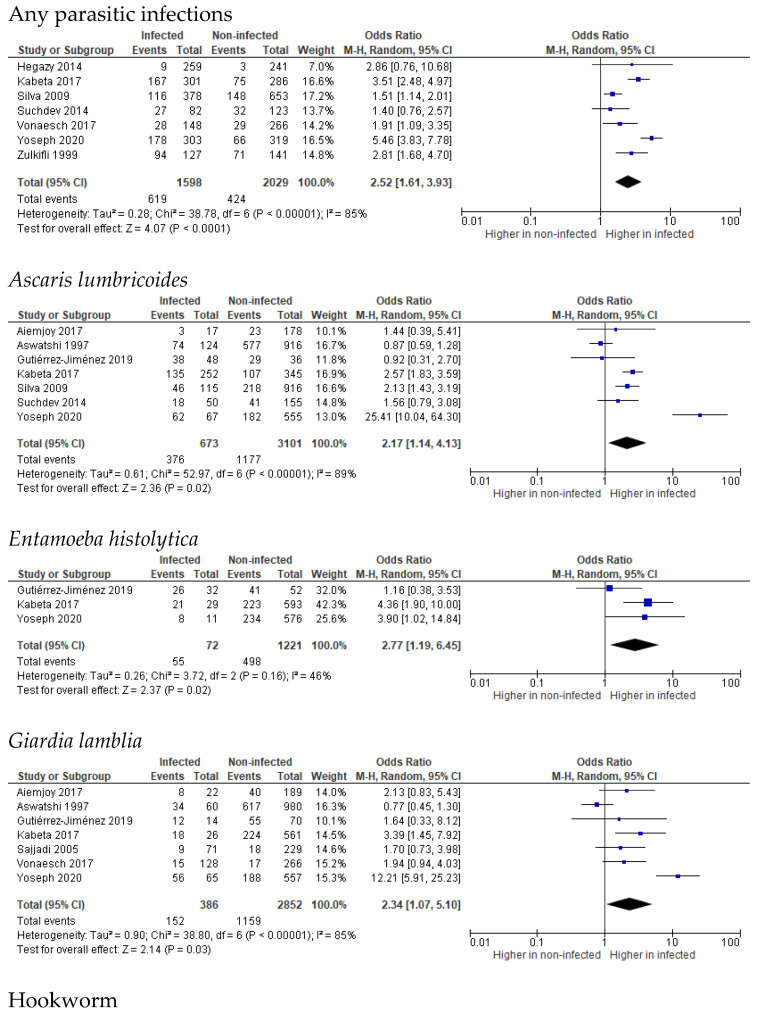

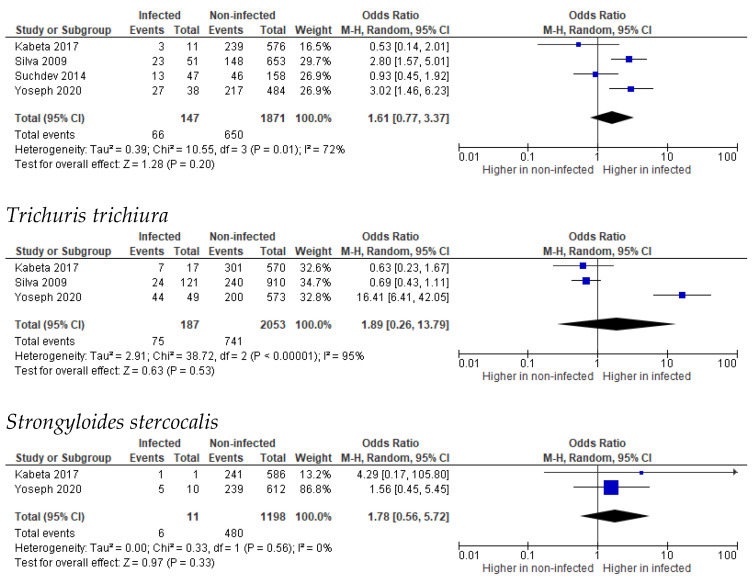
Forest plots of parasitic infections and stunting effect size [37,39,40,41,42,43,45,46,47,48,49].

Stunting is the most reported nutritional status in these studies. Stunting prevalence was reported to be significantly higher in children infected with unspecified intestinal parasites by five studies [40,41,45,47,49] with 1.51- to 5.46-fold increased risks, while two studies found no significant difference between infected and non-infected children [39,48]. Effect size analysis showed 2.52-times (95% CI 1.61, 3.93 (*p* < 0.0001), I^2^ = 85% (*p* < 0.00001)) higher odds of stunting cases in infected children (Figure 2). Several studies reported the significance of specific parasite manifestations toward stunting, including *A. lumbricoides* in three studies [41,43,45] from eight reporting studies [37,41,43,45,46,47,48,49] with a 25.41-, 2.46-, and 2.13-fold increased risk, respectively, *E. histolytica* in one study [41] from three reporting studies [37,41,45] with a 4.36-fold increased risk, *G. lamblia* in two studies [41,45] from seven reporting studies [36,38,40,41,43,45,46] with a 12.31- and 3.39-fold increased risk, Hookworm in one study [41] from three reporting studies [41,45,47] with a 16.41-fold increased risk, and *T. trichiura* in two studies [41,47] from four reporting studies [41,45,47,48] with 3.02- and 2.52-fold increase risks. Effect size analysis showed significant odds of stunting in children infected with *Ascaris lumbricoides* (OR 2.17 (95% CI 1.14, 4.13), *p* = 0.02, I^2^ 89% (*p* < 0.00001))*, Entamoeba histolytica* (OR 2.77 (95% CI 1.19, 6.45), *p* = 0.02, I^2^ 46% (*p* = 0.16))*,* and *Giardia lamblia* (OR 2.34 (CI 1.07, 5.10), *p* = 0.03, I^2^ = 85% (*p* < 0.00001)), as shown in Figure 2. Meanwhile, the overall effect sizes for hookworm, *T. trichiura,* and *S.stercocalis* were not significant. High heterogeneity between studies was noted (I^2^ ranging from 72% to 95%). *E. vermicularis* [45], *H. nana* [45], and *Taenia* spp. [41] were only reported in one study each with no significant difference between infected and non-infected children.

### 3.3. Wasting

**Figure 3:**
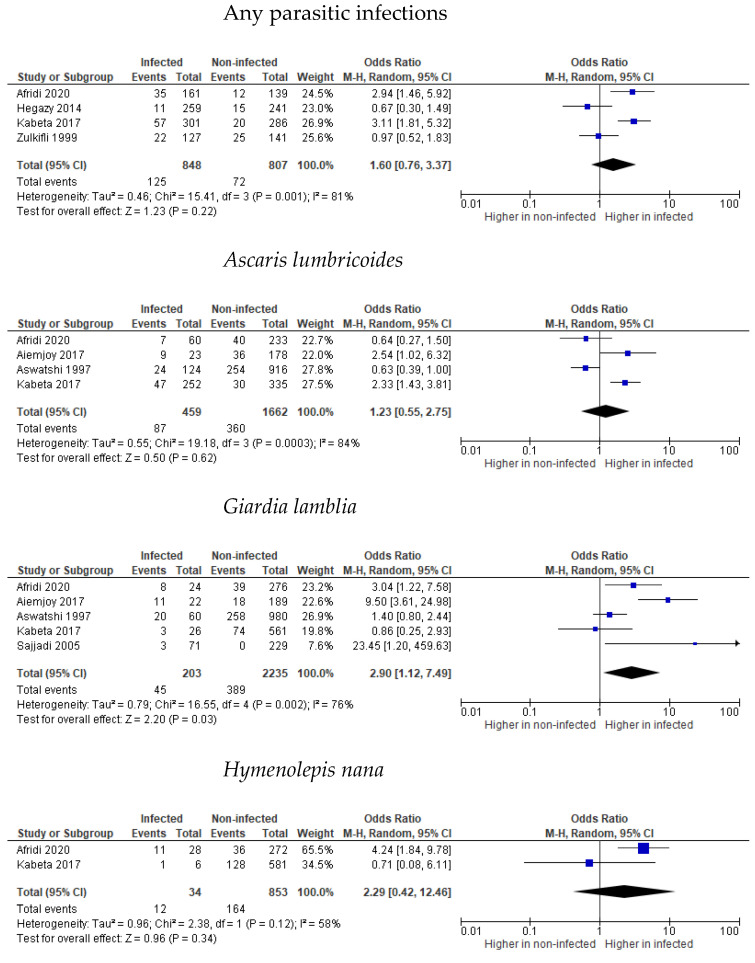
Forest plots of parasitic infections and wasting effect size [36,39,42,43,45,46,49].

There were notably higher numbers of infected children who experienced wasting compared to non-infected children, as reported in two [42,45] out of four reporting studies [39,42,45,49]. Afridi (2020) and Kabeta (2016) reported wasting prevalence rates of 2.94 and 3.11 in children with any intestinal parasites [42,45]. However, based on the effect size analysis, wasting was not related to parasitic infections (*p* = 0.22), as can be observed in Figure 3. High heterogeneity between studies was noted (I^2^ = 81%, *p* = 0.001). Kabeta et al. (2016) reported a higher prevalence of wasting in children with different intestinal parasitic infections, which were caused by *A. lumbcricoides,* hookworm, and *T. trichiura,* with 2.33-, 2.98-, and 3.94-fold increased risks, respectively, while no significance was found in children with *E. histolyca, E. vermicularis*, *H. nana*, and *S. stercocalis* infections. Aiemjoy (2017) found a significant risk of wasting after adjusting the analysis for household income with 2.54 higher odds [36]. In contrast, Afridi (2020) and Aswatshi (1997) found no significant differences between *A. lumbricoides* and the prevalence of wasting [42,43]. Effect size analysis of the studies showed no differences in the wasting prevalence in infected and non-infected children (*p* = 0.62), as shown in Figure 3, with high heterogeneity between studies (I^2^ = 84%, *p* = 0.0004). In contrast to Kabeta (2017), Afridi (2020) found 4.24 higher odds of wasting prevalence in children with *H. nana* infection [42]. Effect size analysis showed 2.29 higher odds of wasting in children with *H. nana* infection; however, the result was not statistically significant (*p* = 0.34) (Figure 3). Afridi (2020), Saijadi (2005), and Aiemjoy (2017) found the wasting prevalence of children infected with *G. lamblia* to be significant, with 3.04, 23.45, 9.50 higher odds, respectively, while Ashwatsi (1997) and Kabeta (2017) did not find any difference [36,42,43,44,45,46]. Size effect analysis showed 2.90 higher odds (95% CI 1.12, 7.49, *p* = 0.03) of wasted children in relation to *G. lamblia* infection. High heterogeneity was noted (I^2^ = 76%, *p* = 0.002).

### 3.4. Underweight

**Figure 4:**
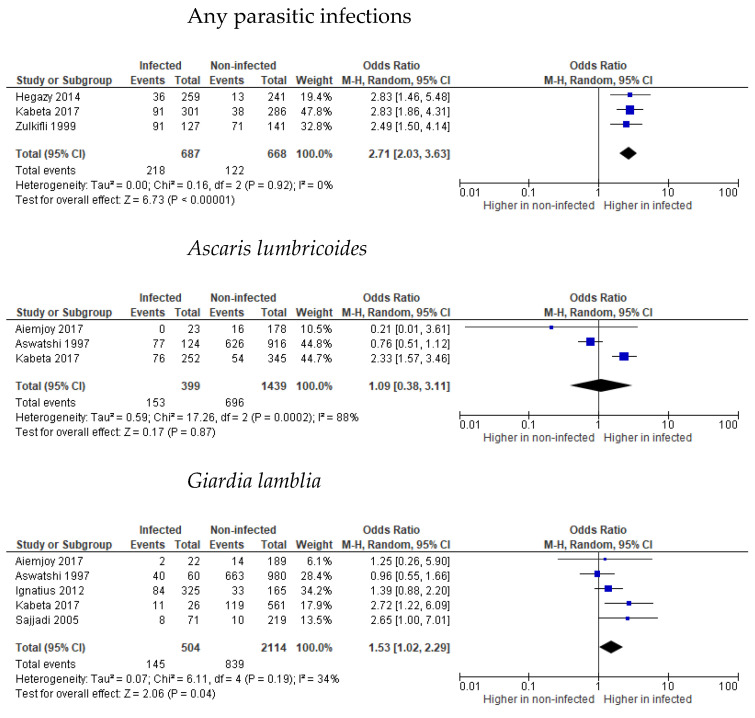
Forest plots of parasitic infections and underweight effect size [36,39,43,44,45,46,49].

Three studies reported a 2.49–2.96 higher prevalence of underweight in children infected with unspecified intestinal parasites [39,45,49]. The magnitude of the effect of parasitic infections on underweight children was 2.71-times higher (95% CI 2.03, 3.63, *p* < 0.00001, I^2^ = 0% (*p* = 0.92)). The studies were in good agreement and no heterogeneity was noted (Figure 4). One [45] out of three studies [36,43,45] found a 2.25-times higher prevalence of underweight in children infected with *A. lumbricoides*. The overall effect size was not significant (*p* = 0.87), but demonstrated significantly high heterogeneity (I^2^ = 88%, *p* = 0.00002) (Figure 4). Underweight prevalence in children with *G. lamblia* infection was higher with a 2.72- and 2.76-times higher risk, as reported in two studies, respectively [45,46], meanwhile three studies did not find any significance [36,43,44]. Overall effect size showed a 1.53-times (95% CI 1.02, 2.29, *p* = 0.04) increase in underweight prevalence in infected children. Low heterogeneity between the studies was noted (I^2^ = 34%, *p* = 0.19), as shown in Figure 4. No significant prevalence of *E. vermicularis, E. histolyca, H. nana, S. stercocalis*, hookworm or *T. trichiura* was noted in underweight children, as reported by Kabeta [45].

### 3.5. Undernutrition

The incidence of undernutrition was found to be very high in infected children with up to 2.58- and 4.28-times higher prevalence, as reported in two studies [38,50]. Specifically, Haratipour (2016) found a very high incidence of undernutrition in children with *Enterobius vermicularis* infection, with a 7.64-fold increased risk compared to non-infected children [38].

### 3.6. Risk Factors Related to Parasitic Infections

Several risk factors related to parasitic infection, as reported by some of the reviewed studies, are summarized in Table 3. The most reported and significant risk factor associated with a higher degree of parasitic infections is socioeconomic background, as reported by five studies [36,38,42,47,50]. Children born from a family in a rural area, with a low household income and with an illiterate father and/or mother are at a higher risk of acquiring infections. House crowding, water sanitation, and personal hygiene are also known as other risk factors. A higher risk of infections was found in children aged > 12 months. Breastfeeding reduced the risk of infection.

## 4. Discussion

Parasitic infection has been reported as one of the contributing factors to undernutrition in preschool children worldwide, with 230 million (43%) estimated cases in the developing world [52]. Although children under the age of five are vulnerable to parasitic infection and are considered to be in their “golden years,” research on intestinal infection in this age group is limited compared to school-aged children. Most anthelmintic campaigns currently target school-aged children that are familiar with the effects of worm infections. From an operational standpoint, this is a “more cost-effective” age group that should receive prioritized treatment. However, this “preventive chemotherapy” policy is based on the assumption that parasitic infection is unimportant during the first five years of life. Furthermore, it is believed that deworming school-aged children, who were reported to be more severely infected, has a significant impact on community-wide parasite transmission [53].

According to the current review, stunting, wasting, underweight, and undernutrition are more common in children with parasitic infections. Ascariasis, hookworm infection, trichuriasis, and giardiasis are four of the most commonly reported infections. This finding is consistent with a previous review by Fauziah et al. (2022), which included 17 studies from Latin America, Southeast Asia, and Africa. They reported that three of the most common soil-transmitted helminths found in children under five were *Ascaris lumbricoides*, hookworm, and *Trichuris trichiura*. They also discovered that stunted children were at higher risks of STH infections than those with normal growth [54].

*Ascaris lumbricoides* is the most commonly reported parasite associated with stunting and underweight. The host becomes infected with Ascaris through the faecal–oral route. The infection begins when the infective eggs that contain L3 larvae are consumed. The ova will hatch in the small intestine, and the larvae will invade the intestinal mucosa, before migrating via portal blood to the trachea. Larvae then enter the alveolar space, move to the pharynx, and are swallowed, eventually returning to the small intestine on days 8–10 after infection and reach sexual maturity on day 24. Adult worms can live in the intestines for up to a year. Female worms can lay between 200,000 and 300,000 eggs per day. Fertilized ova are released into the environment via faeces and can survive in the soil for up to 15 years. The three-layer shell structure of the ova makes it resistant to environmental conditions. High ova production rates and resistance makes ascariasis the most common parasitic infection in the world. Furthermore, because the majority of infections are asymptomatic, they often go undiagnosed [55].

Giardiasis is the second most prevalent infection reported in the studies. It has been linked to an increased risk of stunting, wasting, and being underweight. Giardia infection occurs when the protozoa cyst is consumed through contaminated food, water, hands, or surfaces. After being exposed to the stomach’s acidic environment, the cysts expel two trophozoites into the proximal small intestine, where they feed and absorb nutrients. Trophozoite is the vegetative form that causes diarrhoea and malabsorption symptoms. Trophozoite reproduces through longitudinal binary fission. Trophozoite migrates to the colon and, after being exposed to bile acid, forms a new cyst that is easily released into the environment when passed in the stool. The cyst is inert, allowing it to survive in various environmental conditions [56]. Furthermore, parasites’ resistance to chlorination poses another public health challenge because this parasite can be transmitted through contaminated drinking water [57]. Because of their high survival rate, this parasite is the fourth most common parasite found in malnourished children in this study.

Hookworm is the third most commonly reported parasite related to the prevalence of stunting and wasting. Two major genera, *Nectator americanus* and *Anchylostoma duodenale,* infect humans. Hookworm infection begins when L3 larvae penetrate the skin via percutaneous invasion. Within 1–3 weeks of infection, larvae that penetrate the skin will migrate extra-intestinally through the venous circulation and lungs, before ascending to the trachea and entering the gastrointestinal tract. Larvae will go through two moults before reaching sexual maturity. An adult hookworm’s buccal capsule is armed with cutting plates (*N. amercinanus*) or teeth (*A. duodenale*) that allow it to attach to the intestinal mucosa. Female hookworms can lay between 10,000 (*N. americanus*) and 30,000 eggs (*A. duodenale*) per day. The eggs are passed in the faeces and, if deposited in soil with enough moisture and shade, develop into larvae and hatch. Hookworm larvae can survive outside the host for about six weeks after hatching [58]. Hookworm infection prevalence is also related to children’s behaviour, particularly in rural areas, where children frequently go barefoot and play with soil. Raising buffaloes, pigs, or other animals with mud-bathing habits increases infection risk [59].

*T. trichiura* is also one of the most reported parasites related to stunting and wasting. *Trichuris* infection begins when eggs from contaminated food or water are consumed. Microbial molecular signals induce *T. trichiura* eggs to hatch in the large intestine. Larvae 1 colonize the large intestine crypts and then penetrate the epithelial cells. L1 and L2 larvae are fully enteric, developing within epithelial cells, whereas L3 and L4 larvae protrude partially into the lumen. Patent infections develop over 2–3 months and the parasite can live in the human intestine for 1–8 years. *T. trichiura* has become the third most commonly reported parasite to cause stunting, which can be attributed to its frequent co-infection with *A. lumbricoides*, particularly in endemic areas, where their distributions overlap [60].

*Entamoeba histolytica* has been reported in three studies to be related with stunting incidence. *Entamoeba histolytica* is an invasive enteric protozoan. The ingestion of mature, quadrinucleated cysts in contaminated food or water initiates the infection. In the small intestine, excystation releases motile trophozoites that immediately migrate to the large intestine. Trophozoites form new cysts through binary fission. Both trophozoites and cysts are released in faeces. Due to their protection by their walls, cysts can survive in the environment for weeks. Trophozoites are quickly destroyed once outside the body or by gastric secretions if ingested [61].

The correlation between *Hymenolepsis nana* and wasting has been highlighted in two studies. Afridi highlighted a 4.24-times higher prevalence of wasting in children infected with this parasite [42]. In contrast, Kabeta found no significance of the presence of this parasite with regard to wasting [45]. *H. nana* is a dwarf cestode with a size of 1 to 4 cm long and a 1 mm diameter that infects human and rats. It has a minute scolex with a 0.32 mm diameter, a rhomboidal shape, 4 suckers, and a short rostellum armed with a single row of 20–30 hooklets. It has about 200 segments of proglottides. *H. nana* infection in human begins through ingestion of embryonated eggs or insects that contain cysticercoids The oncospheres are released from the eggs upon ingestion. Oncospheres (hexacanth larvae) enter the villus of the intestine and develop into cysticercoid larvae. When the villus ruptures, the cysticercoids enter the intestinal lumen, evaginate their scoleces, attach to the intestinal mucosa, and mature into adults. When the eggs are released from proglottids through the genital atrium or when proglottids disintegrate in the small intestine, they pass through the stool. Internal autoinfection is an alternative mode of infection in which the eggs release their hexacanth embryo, which penetrates the villus and continues the infective cycle without passing through the external environment. The life span of adult worm is 4–6 weeks and the eggs cannot survive more than 10 days. The low survival rate of the parasite might explain the low prevalence of *H. nana* infection. *H. nana* is a cosmopolitan cestode commonly found in temperate regions [62]. For this reason, *H. nana* infection was found to be higher in the study by Afridi, which was conducted in Pakistan with a temperate climate, compared to Kabeta, which was conducted in Ethiopia with a tropical climate.

*Enterobius vermicularis* has been reported to be related with the significant prevalence of undernutrition by Haratipour et al. (2016) [38], which is in opposition to the findings reported by Kabeta et al. (2017) [45]. *E. vermicularis* infection begins through ingestion of infective eggs via contaminated hands or fomites. Larvae hatch in the small intestine and mature into adults in 15–40 days. Male adult larvae fertilize the female (in the lower ileum) and are excreted without causing symptoms. Females settle in the lower ileum, caecum, appendix, and ascending colon. The gravid worm migrates from the colon to rectal area and lays eggs on the perianal skin at night. The eggs become infective within 6 h at body temperature. Eggs can survive in a cool, moist environment with little ventilation for up to three weeks. However, infectivity declines over time. Eggs do not tolerate heat well, which may explain why enterobiasis is more common in temperate climates rather than tropical climates. This fact may explain the low enterobiasis prevalence in Kabeta’s study (Ethiopia, tropical climate) compared to Haratipour’s study (Iran, temperate climate). *E. vermicularis* is transmitted via the following four ways: (1) direct infection from infected fingernails in the anal and perianal regions; (2) exposure to viable eggs on fomites (clothing, toys, bed, furniture, pet fur, etc.); (3) dust contaminated with embryonated (infected) eggs; (4) retroinfection; migration of larvae into the sigmoid colon and caecum after hatching on the anal mucosa. Although death is extremely uncommon and the cure rate is high, recurrences are common. It is extremely difficult to eradicate pinworms from children, and long-term monitoring is required. Each family member must be treated to completely eradicate the pinworm [63].

In terms of pathophysiology, these parasites cause undernutrition in different ways. *A. lumbricoides* may induce anorexia, nutrient malabsorption, and jejunal mucosal abnormalities. As this parasite lives in the lumen of the small intestine, where digestion and absorption of nutrients occur, parasite–host nutrient competition causes chronic undernutrition. Several studies had shown the relationship between ascariasis and iron-deficiency anaemia (IDA) [64,65]. Animal studies showed that Ascaris impairs glucose–alanine transport in the host intestine. In an Ussing chamber experiment, *Ascaridia galli* inhibited sodium-coupled transport of glucose and alanine. *Ascaris suum*, a pig roundworm, secreted excretion-secretion (ES) antigens, which inhibited jejunum glucose–alanine transport. Cuticle somatic (CSO) antigens influenced Na^+^ /H^+^ exchanger 3 (NHE3), altering intracellular pH and peptide transporter 1 (PepT1) function [66]. Exposure to roundworm antigens also activated the local immune system, specifically Th2 infiltrations and cytokine induction, altering the gut-microbiota balance. The condition complicates the pathogenesis of undernutrition by causing an overgrowth of pathogenic bacteria with bile acid deconjugation on the epithelial surface, resulting in local irritation and damage to the mucosa [67,68]. *A. lumbricoides* can also cause acute manifestations, such as intestinal and biliary tract obstruction with associated complications. The condition is a consequence of earlier *A. lumbricoides* larval migration, which promotes local type 2 inflammatory responses [68].

*Giardia lamblia* resides in the lumen and does not invade the host mucosal barrier. Giardia survives and replicates by utilizing and sequestering nutrients, such as bile and arginine. As a result, Giardia infection is frequently associated with vitamin A and zinc deficiencies because vitamin A is an oil-soluble vitamin that requires bile acid to be released. Giardia evades host inflammatory responses by producing antioxidants, cleaving interleukin-8 (IL-8), depleting arginine via arginine deiminase (ADI), and shifting variant surface protein (VSP) expression. Giardia may affect epithelial cells through cell-cycle arrest, proliferation impairment, tight-junction disruption, and apoptosis induction through direct or indirect mechanisms [69]. Apical junction complex breakdown causes intestinal permeability, allowing various luminal antigens, including microbial factors and food antigens, to enter the sub-epithelial compartment. Trophozoites also cause chloride ion secretion. The accumulation of undigested carbohydrates and ion secretion create an osmotic gradient within the small intestinal lumen, resulting in water loss, intestinal distension, and rapid peristalsis, which eventually causes malabsorptive and hypersecretory diarrhoeal disease. Small intestinal malabsorption is thought to be the primary cause of diarrhoea during Giardia infection, with chloride hypersecretion also contributing to diarrhoeal symptoms [70].

Anaemia is the most common pathophysiology associated with hookworm. Hookworm consumes host erythrocytes via a proteolytic mechanism. Proteases cleave haemoglobin, and the by-product haem is processed by glutathione S-transferase GST-1. However, blood loss is mainly caused by mucosal layer disruption, due to plate teeth attachment. Undernutrition occurs as a result of a moderate-to-heavy infection in which blood loss exceeds the host’s intake and reserves of iron and protein. Kwashiorkor, a severe form of undernutrition, can result from hypoalbuminaemia and hypoproteinaemia. Due to severe anaemia, chlorosis (yellowish skin colour) may also occur. Chronic infection is a result of tolerogenic pathway activation induced by the worm’s excretory–secretory products. This pathway induces dendritic cells (DC) to reduce their level of activation and co-stimulatory markers, activation of M2 macrophages and Treg cells that have anti-inflammatory properties [58].

The pathogenesis of *Trichuris trichiura* is primarily caused by mucosal damage with endogenous losses, which is related to their burrowing mechanism into the gut epithelial. Although not directly related to the nutrient absorption mechanisms, *T. trichiuris* infection is correlated with chronic colitis [60]. *T. trichiuris* infection has also been linked to anaemia and ineffective iron supplementation. The condition is due to blood loss caused by mechanical damage to the intestinal epithelium and the subsequent feeding behaviours. Intestinal damage also causes a loss of appetite, resulting in less food and iron intake [71].

Most *E. histolytica* infections are asymptomatic; nevertheless, invasive intestinal disease can cause cramping, abdominal pain, watery or bloody diarrhoea, and weight loss. Several cases of disseminated extraintestinal manifestation have been reported, such as liver abscess, pneumonia, purulent pericarditis, and cerebral amoebiasis. The pathogenesis of *E. histolytica* is initiated by trophozoite adherence to the colonic epithelium and its lytic abilities. The Gal/GalNAc lectin, which targets galactose and N-acetyl-D-galactosamine residues on the O-linked sugar side chains of mucins, is responsible for adhesion and colonization. Virulence of *E. histolytica* was determined by the presence of the enzyme glycoside hydrolase B-amylase, which removes branched polysaccharides from mucin cells. This allows the trophozoites to penetrate the colonic epithelium and degrade the protective mucous barrier, increasing the risk of metastasis to distant sites. Interactions between E. histolytica and the host’s intestinal flora may mediate pathogenic behaviour, resulting in more virulent strains. Enteropathogenic bacteria can increase the expression of the Gal/GalNAc lectin in E. histolytica trophozoites, leading to increased adhesion capacity and cytopathic effects. The presence of certain gut bacteria increased the production of proinflammatory cytokines, causing additional epithelial damage and facilitating trophozoite invasion [61].

The majority of *Hymenolepis nana* infections are asymptomatic. In severe infections, H. nana causes mechanical irritation of the intestine and may cause irritability, diarrhoea, abdominal pain, sleep disturbances and anal and nasal pruritus due to the release of toxic metabolites [62].

*E. vermicularis* infection is commonly asymptomatic. The most common symptom is perianal pruritus, which can lead to excoriations and bacterial superinfection. Teeth grinding, enuresia, insomnia, anorexia, irritability, and abdominal pain are also some of the symptoms. *E. vermicularis* larvae are frequently found within the appendix during appendectomy, but it is believed that the presence of this parasite in the appendix is a coincidence. Very few cases of eosinophilic colitis associated with *E. vermicularis* larvae have been reported [63]. Although the infection is commonly asymptomatic, a study by Celiksoz et al. (2010) showed that children with *E. vermicularis* infection tend to have lower weights and heights and lower school success [72]. Daoody and Al-Bazzaz (2020) found significantly lower serum total protein and iron levels in infected children [73]. However, to date, no studies have found an association between enterobiasis and anaemia [74,75].

Growth occurs in the following four interconnected phases throughout development: foetal, infant, childhood, and pubertal phases. Maximum growth velocity is typically achieved between birth and 6 months or during the infancy period, a critical period for long-term cognitive development. Linear growth occurs from the ages of 6 to 24 months. In this phase, demand for nutrients is high and if faced with a poor nutrient environment, children will be stunted [76]. During short or long periods of insufficient caloric intake, the body will first use carbohydrate stores for energy, followed by protein and fat. Protein is the final resource because the body requires this macronutrient to function normally. The liver and pancreas are the first organs to suffer from starvation. After a meal, monosaccharides are converted to glucose in the liver and stored as glycogen. When blood sugar levels fall, the pancreas releases insulin, causing the liver to break down glycogen reserves and release glucose into the bloodstream. When glycogen stores are depleted, muscle tissue protein and lactate breakdown occur. However, this method will not suffice to meet long-term starvation needs. Nitrogen released from amino acid substrates produces urea, which in large quantities can cause fat reserves to be used as energy. Unfortunately, protein breakdown is not an efficient energy mining method, due to the fact that the energy consumed in the process nearly equals what is produced [77]. Both wasting and stunting are associated with fat loss and muscle mass loss. Fat-produced hormones play an important role in immune function and bone growth, which could explain the reduced linear growth in relation to low WHZ scores. Some evidence suggests that stunted children, in addition to muscle and fat loss and hormonal imbalances, suffer from deficits in the form of small organ sizes [78].

This systematic review discovered a link between malnutrition and parasitic infection. Malnutrition can result from parasitic infections, both acute and chronic. Acute malnutrition in the form of underweight can be caused by repeated diarrhoeal episodes, which are common during *G. lamblia* infection. Exposure to intestinal parasites causes chronic activation of gut immune cells, resulting in environmental enteric dysfunction. Gut mucosal cell villous atrophy, crypt hyperplasia, increased permeability, and inflammatory cell infiltration are all symptoms of EED. Chronic EED causes gut immune response imbalance and altered gut microflora, both of which play important roles in enteric immunity. EED and repeated diarrhoea cause nutrient malabsorption and, together with the intake of poor-quality food, result in growth retardation in the form of wasting and stunting [76].

During chronic infections, extensive breakdown of protein occurs in malnourished children. Protein–energy undernutrition (PEU), or primary undernutrition, contributes to a weakened immune system, which can be explained by the following two mechanisms: (1) essential nutrient deprivation that affects the production and function of immune cells; (2) adaptation to nutrient deprivation that results in qualitative changes in immune system metabolism. Physiological changes in the immune system have been thoroughly studied and are summarized as follows. For the adaptive immune system, PEM caused (1) low percentage of rosette-forming cells (indicator T-lymphocyte number); (2) increased percentage of null cells (indicator of impaired production of thymic hormones); (3) fewer T-cells and decreased helper-suppressor cell ratios (T4/T8); (4) decreased lymphokine and monokine production (TNF, prostaglandin, E_2_, IL-I and fibronectin). For the innate immune system, PEM caused (1) decreased complement C3 and total haemolytic activity; (2) decreased opsonic function of plasma and intracellular killing capacity of polymorphonuclear leukocytes; (3) decreased serum toxaemic factor; (4) impaired production of gamma interferon and IL-1 and IL-2; (5) phagocyte dysfunction; 6) decreased antibody affinity; (7) impaired secretory IgA antibody response; and (8) decreased response of acute-phase reactants [77]. These conditions increase the risk of acquiring infections and their severity.

A systematic review by Thurstan et al. (2022) showed the relationship between wasting and stunting. Wasted children were most likely to also be stunted. One hypothesis for this condition is that children experience halted linear growth until they regain a healthy body weight in response to weight faltering [78]. However, sometimes, underweight and wasting are not related to stunting. Brind et al. (2015) highlighted the coexistence of high body fat in stunted children. They propose that high fat reserves alone are insufficient to support linear growth, and that a lack of nutrients such as zinc, sulphur, phosphorous, vitamins D, C, and K, and copper—nutrients that are required for bone growth and lean tissue synthesis—may explain the association between stunting and normal or increased fat reserves. Leptin deprivation, as a result of previous wasting, may also have an effect on bone growth [79]. Our review found that parasitic infection was related to stunting and being underweight, but not wasting. The possible explanation is that children were both stunted (low HAZ) and underweight (low WAZ); hence, the wasting value (low WHZ), which is the division between weight and height, becomes normal.

Parasitic infections are more common in children from families with low socioeconomic backgrounds. This could be due to their inability to provide nutritious food, a lack of knowledge regarding childcare delivery, inadequate sanitation facilities (i.e., the availability of clean water), and insufficient access to healthcare facilities. Intestinal parasitic infections are also linked to personal hygiene because the majority of infections occur through the faecal–oral route. As shown in Table 3, some hygiene risk factors include playing with soil and not washing hands. As most people in rural areas do not have separate housing for their animals, soil can become contaminated with animal faeces. Hand-to-mouth and exploratory behaviour in children may increase the likelihood of infection. Overcrowding in the home with many family members is also a factor, as it leads to intra-family transmission from close contacts, such as when feeding and bathing infants [78,80]. Interestingly, the presence of siblings under five years old is also a risk factor. We conclude that children under 5 years old are a potent carrier of intestinal parasite infection. Breastfeeding practice reduced the risk of parasitic infections. Breast milk colostrum contains growth factors and cytokines, such as epidermal growth factor (EGF), nerve growth factor (NGF), insulin-like growth factor (IGF), TNF-α, transforming growth factor-α (TGF-α), basic fibroblast growth factor (bFGF), transforming growth factor-β (TGF-β), granulocyte colony-stimulating factor (GCSF), in addition to interleukins IL-1β, IL-6, IL-8 and IL-10, prostaglandin and milk cortisol that stimulate growth and strengthen gut mucosal integrity. Mucin and immunoglobulin A (IgA) in breast milk can neutralize the penetrative action of some geohelminths and protozoans. Infections are less prevalent in children aged under 12 months, as during this age, children are often still exclusively breastfed [81].

There are inconsistencies in the findings of the studies examined here due to several factors. First, the small sample size reduces the statistical power and effect size flexibility. Second, there might be associated risk factors that are not analysed in the studies, such as antenatal care, breastfeeding practice, previous anthelminthic therapy and previous supplementary acute malnutrition dietary interventions, which may be related to children’s immunity, growth, and development [36,37,38,39,40,41,42,43,44,45,46,47,48,49,50]. Third, parasitic infection severity is not examined or reported in the reviewed studies, which is related to the degree of undernourishment. Fourth, geographical and seasonal variations may be associated with the most prevalent infection in the study area. In a study in Mozambique, for example, there was inter-province variability in common parasitic infections. In the Province of Sofala, *Cryptosporidium* spp., *T. trichiura*, and *A. lumbricoides* are the three most common parasites. Meanwhile, *G. lamblia* was the most commonly identified parasite in Nampula. More parasites were found during the rainy season than during the dry season. Peak infection of *Cryptosporidium* spp. was observed during the wet period, whereas *G. lamblia* was more prevalent during the dry period [82]. Hookworm infection is more common in plantation and agricultural areas, where farmers and their children typically work barefoot in muddy conditions, creating an ideal environment for hookworm development [55,59]. Last, most studies included in the review are cross-sectional studies, which lack the temporal association between exposure and outcome. As a result, we cannot draw a certain conclusion regarding parasitic infection causality toward undernourishment.

**Figure 5:**
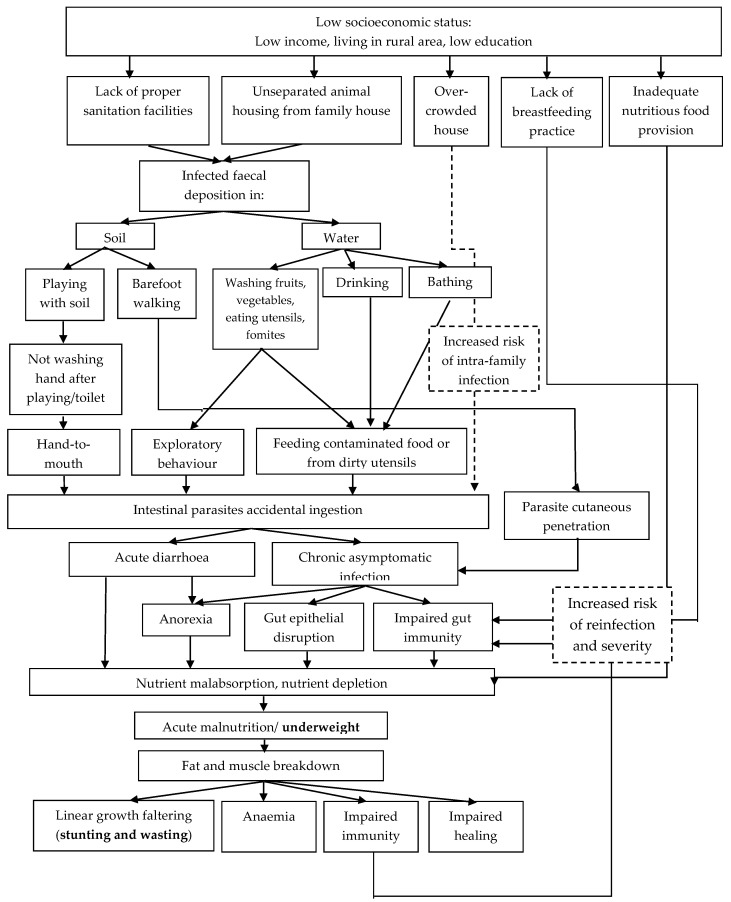
Relationship between low socioeconomic status, parasitic infections, and undernourishment.

We propose a model of the relationship between intestinal parasite infection and undernutrition, as can be observed in Figure 5. Low socioeconomic status (low income, living in a rural area and low education) is associated with a lack of proper sanitation facilities, unattached animal housing from the main house, overcrowding, lack of breastfeeding, and insufficient nutritious food provisions. Faecal contamination in soil and water is caused by a lack of proper sanitation and unseparated animal housing. Accidental ingestion of parasites occurs when children do not wash their hands after playing with soil, put contaminated utensils or fomites in their mouth, drink contaminated water or accidentally drink while bathing, and eat uncooked foods that have been washed with contaminated water. Infections can also occur via cutaneous penetration when children walk barefoot on contaminated soil. Anorexia, gut epithelial disruption, and impaired gut immunity are all symptoms of parasitic infection. Following parasitic infections, children can experience nutrient malabsorption and depletion and become underweight. Undernourishment can also be caused by a lack of high-quality food. Chronic malnutrition and recurrent infection force the body to break down fat and muscle in order to provide adequate energy and achieve functionality. This condition causes linear growth failure (stunting and wasting), anaemia, impaired immunity, and impaired healing, creating a vicious cycle of increased risk of reinfection and severity. Lack of breastfeeding is associated with weakened immunity, which increases the likelihood and severity of reinfection. Overcrowding in the home also increases the risk of intra-family reinfection.

More evidence is needed to confirm the relationship between a specific type of parasite that causes forms of undernutrition. We can conclude that *A. lumbricoides* and *G. lamblia* are the main culprits of undernutrition in children under five years old. Improvements in water and sanitation, health education, and drug treatment may help break the transmission cycle, and effective drugs will reduce morbidity. Deworming (mass treatment) using albendazole, mebendazole, and ivermectin is an effective parasitological cure that leads to significant reductions in egg excretion and is effective against *Ascaris lumbricoides* infection. These drugs are safe for treating children and adults who have been diagnosed with ascaris [83]. Although deworming programs are effective against STH infections, mass deworming alone is insufficient to improve growth, cognition, school performance or school attendance of children living in endemic areas, as shown by the network meta-analysis carried out by Welch et al. (2016). This policy is only effective at improving weight in cases of schistosomiasis [84]. In addition to reconsidering mass deworming programs, additional policy options for improving child health and nutrition in worm-endemic areas should be investigated. These include nutritional intervention programs and the need to invest in interventions that address the basic determinants of worm infestations, such as poverty, living conditions, sanitation, and inequity.

Giardiasis is a water-borne infectious disease. Giardia can be found in wastewater from wastewater treatment plants. Many of these organisms are difficult to remove, making effective wastewater treatment difficult. Because of their resistance to chemical disinfection, Giardia may remain after most contaminants have been removed. Giardia cyst oocysts are extremely resilient and can survive in water for months. Effective Giardia cyst removal from water can be achieved by properly functioning conventional filters when the water is effectively treated through coagulation, flocculation and settling prior to filtration. Currently, many membrane filter technologies are being developed to achieve effective cyst removal [85]. A systematic review by Gera et al. (2018) showed that hygiene interventions have little or no effects on most anthropometric parameters. Improvement in water supply and quality was associated with slightly higher weight-for-age Z-scores, but no significant impact on other anthropometric parameters or infectious morbidity was reported. Improvement in sanitation had a variable effect on the anthropometry and infectious morbidity. Combined water, sanitation and hygiene interventions improved the height-for-age Z scores and decreased the risk of stunting by 13%. Any WASH intervention (considered together) resulted in a lower risk of underweight (RR 0.81; 95% CI 0.69, 0.96), stunting (RR 0.77; 95% CI 0.68, 0.86) and wasting (RR 0.12, 0.85).In addition to local management control strategies that involve improved infrastructure for both drinking water and sewage systems, education of society to improve personal hygiene and sanitation has been linked to a lower incidence of giardiasis [86].

In comparison to studies that focus on specific parasites, more studies agree that there is a link between the prevalence of undernutrition and unspecified parasitic infection. This suggests that mixed infections could be a more potent cause of undernutrition. Education of personal hygiene and sanitation, caregiving, deworming programs, nutrient supplementary programs and regular evaluation of infection and nutritional status might control parasite infection and improve linear growth.

## 5. Conclusions

According to the findings of this study, helminthic parasitic infections can occur very early in life and cause significant growth retardation (stunting, wasting, and underweight). It is important to understand the prevalence and effects of infection in these vulnerable preschool-age groups in order to effectively implement therapeutic interventions and prevention controls. More evidence is needed to confirm the current findings, and future research should take geographical, seasonal, socioeconomic, behavioural, and deworming program factors into account. Analysis of the specific type of infecting parasite allows health practitioners to focus more on designing appropriate and targeted treatment. Finally, if parasite transmission is to be completely eradicated, control programs should target younger, usually asymptomatic, age groups to reduce environmental contamination caused by egg dispersal.

## Figures and Tables

**Table 1:** Prevalence of intestinal parasitic infection in the included studies on nutritional status.

Parasite	Study (Year) Country	Stunting (HAZ < −2SD)				Wasting (WHZ < −2SD)				Underweight (WAZ < −2SD)				Undernutrition (HAZ, WHZ, WAZ < −2SD)			
		Infected	Non-Infected	Crude OR (95% CI), *p*-Value	Adjusted OR (95% CI), *p*-Value	Infected	Non-Infected	Crude OR (95% CI), *p*-Value	Adjusted OR (95% CI), *p*-Value	Infected	Non-Infected	Crude OR (95% CI), *p*-Value	Adjusted OR (95% CI), *p*-Value	Infected	Non-Infected	Crude OR (95% CI), *p*-Value	Adjusted OR (95% CI), *p*-Value
All (not specified)	Doni (2015) [50] Şanlıurfa, Turkey													42/58	19/50	4.28 (1.90, **0.0004**	N/A
	Haratipour (2016) [38] Shahroud, Iran													87/649	68/1201	2.58 (1.85, 3.60), **<0.00001**	N/A
	Hegazy (2014) [39] Damanhur City El-Behera Governorate, Egypt	9/259	3/241	2.98 (0.80, 11.12), 0.100	N/A	11/259	15/241	0.70 (0.31, 1.55), 0.38	N/A	36/259	13/241	2.96 (1.53, 5.72), **0.001**	N/A				
	Vonaesch (2017) [40] Bangui, Central African Republic	28/148	29/266	1.91 (1.09, 3.35), **0.025**	0.370 *												
	Yoseph (2020) [41] Boricha Woreda, Southern Ethiopia	178/303	66/319	5.46 (3.83, 7.78), **<0.00001**	N/A												
	Afridi (2020) [42] Skardu, Pakistan					35/161	12/139	2.94 (1.46, 5.92), **0.003**	N/A								
	Kabeta (2017) [45] Hawasa Zuria, South Ethiopia	167/301	75/286	3.51 (2.48, 4.97), **<0.00001**	N/A	57/301	20/286	3.11 (1.81, 5.32), **<0.0001**	N/A	91/301	38/286	2.83 (1.86, 4.31), **<0.00001**	N/A				
	Silva (2009) [47] Itinga, Vale of Jequitinhonha, Brazil	116/378	148/653	1.51 (1.14, 2.01), **0.0005**	N/A												
	Suchdev (2014) [48] Kibera, Nairobi, Kenya	27/82	32/123	1.40 (0.76, 2.57), 0.290	N/A												
	Zulkifli (1999) [49] Kelantan, Malaysia	94/127	71/141	2.81 (1.68, 4.70), **<0.00001**	N/A	22/127	25/141	0.97 (0.52, 1.83), 0.93	N/A	91/127	71/141	2.49 (1.50, 4.14), **0.0004**	N/A				
*Ascaris lumbricoides*	Aiemjoy (2017) [36] Amhara, Ethiopia	3/23	22/178	1.06 (0.29, 3.88), 0.93	1.44 (0.39, 5.41), 0.59 ^+^	3/23	17/178	1.42 (0.38, 5.28), 0.60	2.54 (1.02, 6.32), **0.045** ^+^	0/23	16/178	0.21 (0.01, 3.61), 0.28	N/A				
	Gutiérrez-Jiménez (2019) [37] Chiapas Highlands, Mexico	38/48	29/36	0.92 (0.31, 2.70), 0.88	N/A												
	Yoseph (2020) [41] Boricha Woreda, Southern Ethiopia	62/67	182/555	25.41 (10.04, 64.30), **0.00001**	N/A												
	Afridi (2020) [42] Skardu, Pakistan					7/60	40/233	0.64 (0.27, 1.50), 0.30	N/A								
	Aswatshi (1997) [43] Lucknow, India	74/124	577/916	0.87 (0.59, 1.28), 0.47	N/A	24/124	254/916	0.63 (0.39, 1.00) 0.05	N/A	77/124	626/916	0.76 (0.51, 1.12), 0.160	N/A				
	Kabeta (2017) [45] Hawasa Zuria, South Ethiopia	135/252	107/345	2.46 (1.75, 3.45), **<0.00001**	N/A	47/252	30/335	2.33 (1.43, 3.81), **0.0007**	N/A	76/252	54/345	2.25 (1.51, 3.34), **<0.00001**	N/A				
	Silva (2009) [47] Itinga, Vale of Jequitinhonha, Brazil	46/115	218/916	2.13 (1.43, 3.19), **0.0002**	N/A												
	Suchdev (2014) [48] Kibera, Nairobi, Kenya	18/50	41/155	1.56 (0.79, 3.08), 0.20	N/A												
	*Zulkifli* (1999) [49] Kelantan, Malaysia													34/48	161/220	0.89 (0.45, 1.77), 0.74	N/A
*Cryptosporidium sp.*	Afridi (2020) [42] Skardu, Pakistan					9/42	38/258	1.58 (0.70, 3.56), 0.27	N/A								
*Entamoeba* *histolytica*	Gutiérrez-Jiménez (2019) [47] Chiapas highland, Mexico	26/32	41/52	1.16 (0.38, 3.53), 0.79	N/A												
	Yoseph (2020) [41] Boricha Woreda, Southern Ethiopia	21/29	223/593	4.36 (1.90, 10.00), **0.0005**	N/A												
	Kabeta (2017) [45] Hawasa Zuria, South Ethiopia	8/11	234/576	3.90 (1.02, 14.84), 0.05	N/A	1/11	76/576	0.66 (0.08, 5.21), 0.69	N/A	5/11	125/576	3.01 (0.90, 10.01), 0.07	N/A				
*Enterobius vermicularis*	Kabeta (2017) [45] Hawasa Zuria, South Ethiopia	2/6	240/581	0.71 (0.13, 3.91), 0.69	N/A	1/6	76/581	1.33 (0.15, 11.53), 0.80	N/A	1/6	129/581	0.70 (0.08, 6.05), 0.75	N/A				
	Haratipour (2016) [38] Shahroud, Iran													69/230	86/1620	7.64 (5.36, 10.91), **<0.00001**	N/A
*Giardia lamblia*	Aiemjoy (2017) [36] Amhara, Ethiopia	4/22	21/189	1.78 (0.55, 5.75), 0.34	2.13 (0.83, 5.43) 0.11^+^	5/22	15/189	3.41 (1.10, 10.54), **0.03**	9.50 (3.61, 24.98), **<0.0001** ^+^	2/22	14/189	1.25 (0.26, 5.90), 0.78	1.26 (0.34, 4.62), 0.76 ^+^				
	Vonaesch (2017) [40] Bangui, Central African Republic	15/148	17/266	1.65 (0.80, 3.41), 0.175	0.801 *												
	Yoseph (2020) [41] Boricha Woreda, Southern Ethiopia	56/65	188/557	12.21 (5.91, 25.23), **<0.00001**	N/A												
	Afridi (2020) [42] Skardu, Pakistan					8/24	39/276	3.04 (1.22, 7.58), **0.02**	N/A								
	Aswatshi (1997) [43] Lucknow, India	34/60	617/980	0.77 (0.45, 1.30), 0.33	N/A	20/60	258/980	1.40 (0.80, 2.44), 0.240	N/A	40/60	663/980	0.96 (0.55, 1.66), 0.870	N/A				
	Kabeta (2017) [45] Hawasa Zuria, South Ethiopia	18/26	224/561	3.39 (1.45, 7.92), **0.005**	N/A	3/26	74/561	0.86 (0.25, 2.93), 0.81	N/A	11/26	119/561	2.72 (1.22, 6.09), **0.010**	N/A				
	Gutiérrez-Jiménez (2019) [38] Chiapas Highlands, Mexico	12/14	55/70	1.64 (0.33, 8.12), 0.55	N/A												
	Sajjadi (2005) [46] Marvdhast, Iran	9/71	18/229	1.70 (0.73, 3.98), 0.22	N/A	3/71	0/229	23.45 (1.20, 459.63), 0.04	N/A	8/71	10/219	2.78 (1.05, 7.34), **0.04**	N/A				
	Ignatius (2012) [44] Butare and Huye, Rwanda									84/325	33/165	1.39 (0.88, 2.20), 0.15	N/A				
Hookworm	Yoseph (2020) [41] Boricha Woreda, Southern Ethiopia	44/49	200/573	16.41 (6.41, 42.05) **<0.00001**	N/A												
	Kabeta (2017) [45] Hawasa Zuria, South Ethiopia	7/17	301/570	0.63 (0.23, 1.67), 0.350	N/A	5/17	72/570	2.88 (0.99, 8.42), **0.045**	N/A	7/17	301/570	0.63 (0.23, 1.67), 0.350	N/A				
	Silva (2009) [47] Itinga, Vale of Jequitinhonha, Brazil	24/121	240/910	0.69 (0.43, 1.11), 0.12	N/A												
	Zulkifli (1999) [49] Kelantan, Malaysia													8/10	187/258	1.52 (0.31, 7.32), 0.60	
*Hymenolepsis nana*	Kabeta (2017) [45] Hawasa Zuria, South Ethiopia	4/6	238/581	2.88 (0.52, 15.86), 0.220	N/A	1/6	128/581	0.71 (0.08, 6.11), 0.750	N/A	2/6	128/581	1.77 (0.32, 9.77), 0.510	N/A				
	Afridi (2020) [42] Skardu, Pakistan					11/28	36/272	4.24 (1.84, 9.78), **0.0007**	N/A								
*Strongyloides sterocalis*	Yoseph (2020) [41] Boricha Woreda, Southern Ethiopia	5/10	239/612	1.56 (0.45, 5.45), 0.490	N/A												
	Kabeta (2017) [45] Hawasa Zuria, South Ethiopia	1/1	241/586	4.29 (0.17, 105.80), 0.37	N/A	0/1	77/586	2.19 (0.09, 54.27), 0.63	N/A	0/1	128/586	1.19 (0.05, 29.37), 0.92	N/A				
*Taenia* spp.	Yoseph (2020) [41] Boricha Woreda, Southern Ethiopia	4/8	240/614	1.56 (0.39, 6.29), 0.530	N/A												
*Trichuris trichiura*	Yoseph (2020) [41] Boricha Woreda, Southern Ethiopia	27/38	217/484	3.02 (1.46, 6.23) **0.003**	N/A												
	Kabeta (2017) [45] Hawasa Zuria, South Ethiopia	3/11	239/576	0.53 (0.14, 2.01), 0.350	N/A	4/11	73/576	3.94 (1.12, 13.78), **0.030**	N/A	2/11	128/576	0.78 (0.17, 3.65), 0.75	N/A				
	Silva (2009) [47] Itinga, Vale of Jequitinhonha, Brazil	23/51	148/653	2.52 (1.42, 4.46), **0.002**	N/A												
	Suchdev (2014) [48] Kibera, Nairobi, Kenya	13/47	46/158	0.93 (0.45, 1.92), 0.85													
	Zulkifli (1999) [49] Kelantan, Malaysia													25/37	170/231	0.75 (0.35, 1.58), 0.45	N/A

**^+^** Mixed-effect logistic regression model with random effects for community, adjusted for household income. * OR adjusted for age category, gender, weight-for-height z-score, positive for bacterial culture, N/A: Not Available.

**Table 2:** Prevalence of parasitic infections according to the countries of the included studies.

Parasites	Country											
	Africa					Asia					America	
	Ethiopia (Tropical)	Kenya (Tropical)	Central African Republic (Tropical)	Rwanda (Tropical)	Egypt (Sub-Tropical)	Pakistan (Temperate)	Iran (Temperate)	Turkey (Temperate)	India (Sub-Tropical)	Malaysia (Tropical)	Brazil (Sub-Tropical)	Mexico (Tropical)
Not specified	303/622 (48.71%) [41] 301/587 (51.28%) [45]	82/205 (40.0%) [48]	148/414 (35.77%) [40]		259/500 (51.78%) [39]	161/300 (53.67) [42]	649/1850 (35.08%) [38]	58/108 (53.71%) [50]		127/268 (47.39%) [49]	378/1031 (36.67%) [47]	
*A. lumbricoides*	23/201 (11.44%) [36] 67/622 (10.77%) [41] 252/597 (42.41%) [45]	50/205 (24.39%) [48]				60/293 (20.48%) [42]			124/1040 (11.92%) [43]	48/268 (17.91%) [49]	115/1031 (11.15%) [47]	48/84 (57.14%) [37]
*Cryptosporidium*						42/300 (14.00%) [42]						
*E. histolytica*	29/622 (4.66%) [41] 11/587 (1.87%) [45]											32/84 (38.10%) [37]
*E. vermicularis*	6/587 (1.02%) [45]						230/1850 (12.42%) [38]					
*G. lamblia*	22/210 (10.48%) [36] 65/622 (12.06%) [41] 26/587 (4.43%) [45]		148/414 (35.75%) [40]	325/490 (66.33%) [44]		24/300 (8.00%) [42]	71/300 (23.67%) [46]		60/1040 (5.77%) [43]			14/84 (16.67%) [37]
Hookworm	49/622 (7.88%) [41] 17/587 (2.89%) [45]									10/268 (3.73%) [49]	121/1031 (11.73%) [47]	
*H. nana*	6/587 (1.02%) [45]					11/300 (3.67%) [42]						
*S. sterocalis*	10/622 (1.61%) [41] 1/587 (1.70%) [45]											
*Taenia* spp.	8/622 (1.29%) [41]											
*T. trichiura*	38/622 (6.11%) [41] 11/587 (1.87%) [45]	50/205 (24.39%) [48]								37/268 (13.81%) [49]	51/1031 (4.97%) [47]	

**Table 3:** Risk factors associated with parasitic infections.

Studies	Risk Factors	Summary of Findings
Afridi (2020) [42]	Socioeconomic background	Most children with parasitic infections belonged to low or low-middle SE groups, with 165/300 (55%) children from low SE, 90/300 (30%) from low-middle SE, 30/300 (10%) from middle SE and 15/300 (5%) from upper middle SE groups, respectively.
Aiemjoy (2017) [36]	Household income (socioeconomic background)	Higher prevalence of *Ascaris lumbricoides* infections was found in children from families with a household income of <1USD/day with a 6.68 (95% CI 1.01, 44.34, *p* = 0.042) prevalence ratio.
Gutierrez-Jimenez (2019) [38]	Rural vs urban municipalities (socioeconomic background)	Intestinal parasitic infections were only found in children that lived in rural areas. No infections were found in urban children. There were contrasting conditions of rural houses compared to urban houses, where the former had earthen floor and no access to potable water.
Silva (2009) [47]	Family members (socioeconomic background)	Children that lived with more than 5 family members were more likely to be infected with *G. lamblia* (OR 1.8 (95% CI 1.2–2.8), *p* = 0.011).
	Number of bedrooms (socioeconomic background)	Children that lived in a house with less than 2 bedrooms were more likely to be infected with *G. lamblia* (OR 1.5 CI (95% 1.1–3.0), *p* = 0.012).
	Have brothers/sisters <5 years old	*G. lamblia* infection was noted to be higher in those with brothers/sisters <5 years old (OR 2.7 (95% CI 1.5–4.5), *p* = 0.000).
	Tap water (sanitation)	Children without access to tap water experienced a 2.1-fold (1.3–3.3, *p* = 0.001) increased risk of acquiring *Gl.lamblia* infection.
	Sewage system (sanitation)	*G. lamblia* infection was more prevalent in children that lived in a house without a sewage system with an odds ratio of 1.8 (1.1–3.1, *p* = 0.030).
Doni (2015) [50]	Maternal education (socioeconomic background)	Higher risk of infection in children with illiterate mother (OR 3.019 (95% CI 1.279–7.130, *p* = 0.012)).
	Paternal education (socioeconomic background)	Higher risk of infection in children with illiterate father (OR 4.954 (95% CI 1.819–13.492, *p* = 0.012)).
	Poor economic situation (socioeconomic background)	Higher odds of infection found in children born into a financially poor family (OR 2.945 (95% CI 1.292–6.717, *p* = 0.010).
	Number of households (6 or above)	Higher risk of infections was found in children living with more than 6 family members (OR 2.865 (95% CI 1.233–6.667), *p* = 0.014).
	Children aged 36 months and below	Increased risk of infection in children younger than 36 months (OR 1.93 (95% CI 1.14–3.29), *p* = 0.036).
	Playing with soil (hygiene)	Higher risk of infections in children playing with soil (OR 4.956 (95% CI 1.856–13.528), *p* = 0.000).
	Not washing hands after toilet (hygiene)	Higher risk of infections in children not washing hands after the toilet (OR 6.369 (95%CI 1.361–2.941), *p* = 0.019).
	Wrong technique of washing hands (hygiene)	Higher infections in children not washing hands after the toilet (OR 6.369 (95%CI 1.361–7.299), *p* = 0.007).
Ignatius (2017) [44]	Age of more than 1 year	Increased risk of *G. lamblia* infections in children aged >1 year with the following odds ratios: 1–2 years: 1.97 (1.08–3.59, *p* = 0.03) 2–3 years: 3.82 (2.06–7.07, *p* <0.0001) 3–4 years: 4.52 (2.65–7.72, *p* <0.0001) 4–5 years: 5.52 (3.15–9.70, *p* < 0.0001).
	Breastfed	Lower risk of infections in breastfed children (OR 0.40 (95% CI 0.32–0.51), *p* <0.0004).
	Number of siblings	Higher risk of infections in children that had more than 4 siblings (OR 3.14 (95% CI 1.87–5.27), *p* = 0.0001).
Yoseph (2020) [41]	Consuming raw vegetables and fruits	Higher risk of infections in children that consumed uncooked vegetables and fruits (OR 2.65 (95% CI 1.6–4.7).
	Absence of sanitation facility	Higher risk of infections in children living without sanitation facilities (OR 2.9 (95% CI 1.6–5.3)).
	Wearing shoes	Higher risk of infections in children not wearing shoes (OR 3.5 (95% CI 2.–5.7).
	Family size	Higher risk of infections in children living with a large family (OR 2.7 (95% CI 1.5–5.0).

## Data Availability

Data are contained within the article or supplementary material. The data presented in this study are available at https://www.mdpi.com/article/10.3390/tropicalmed7110371/s1.

## Supplementary Materials

The following supporting information can be downloaded at: https://www.mdpi.com/article/10.3390/tropicalmed7110371/s1, Table S1: Characteristics of the included study; Table S2: Quality assessment of the study based on Joanna Briggs Institute Cross Sectional Study Appraisal Checklist; Table S3: Quality assessment of the study based on Joanna Briggs Institute Case Control Study Appraisal Checklist.
