# Supplementary material for: Intestinal Parasitic Infection and Nutritional Status in Children under Five Years Old: A Systematic Review
Source: Trop Med Infect Dis. 2022 Nov 12;7(11):371. doi: 10.3390/tropicalmed7110371 (PMC9697828; doi:10.3390/tropicalmed7110371)
Supplement: Supplementary file 1 [file tropicalmed-07-00371-s001.zip › tropicalmed-1989155-supplementary.pdf]

Table S1. Characteristic of the included study

No.	Author (Year) Country	Type of study	Inclusion and Exclusion Criteria	Age at assessment	No. of cohort and control	Intestinal Parasite	Outcome	Assessment method	Reason for Exclusion
**1**	Aiemjoy et al. (2017)	Cross-sectional	**Inclusion:**<br>• study took place in a rural agrarian region in the Goncha Siso Enese District (woreda) of Amhara, Ethiopia, during dry season<br>• children 0–5 years of age<br>•	0-5 years	212 samples were collected from 255 randomly selected children	Helminths: Ascaris lumbricoides, Trichuris trichiura, and hookworm; Protozoan: Giardia lamblia and Entamoeba histolytica/dispar	Prevalence of intestinal parasites<br><br>Anthropometry	Kato-Katz<br><br>HAZ<br>WAZ<br>WHZ	
2	Ajjampur et al. (2011) India	Cross-section	**Inclusion:**<br>•Children lived in semi-urban slum in Vellore, India<br>•Having had at least one episode of cryptosporidial or giardia diarrhoea	<2 years	N = 116<br>History of protozoan diarrhoea (n=84)<br>No history of protozoan diarrhoea (n=32)	Protozoa<br>Giardia<br>Cryptosporidial	Anthropometric growth classified as stunted, underweight, wasted<br><br>Social maturity<br><br>Intelligence	HAZ<br>WAZ<br>WHZ<br><br>Vineland social maturity sclae	

			•Below the age of 2 years<br>•Children with no documented 3cyrptosporidial or giardial infection detected by microscopy (control)<br>•					Seguin Form Board Test2	
3	Caron, *et al.* (2018)	Cohort	**Inclusion:**<br>• children under 5 years of age, in Cambodia (Kratie and Ratanak Kiri)<br>• area with high prevalence of domestic and livestock animals at household level	Children under 5 yrs	639 children<br>- 593 children anthropometric data (Kratie n = 302; Ratanak Kiri n = 291)	*G. duodenalis*	exposure to animal feces,<br><br>morbidity<br><br>anthropometric	directly to the mother during the interview microscopic examination WFA, HFA,	
4	Doni *et al.* (2015)	case–control study	**Inclusion:**<br>• Children of Şanlıurfa, Turkey<br>• The control and case groups of children were compared in	Below 6 yrs old	Case group- children having a Z-score of <−3 SD: N= 50<br><br>Control group- children without any health	*Giardia intestinalis*<br>*Enterobius vermicularis*<br>*Ascaris lumbricoides*<br>*Hymenolepis* nana<br>*Trichuris trichiura*<br>*Escherichia coli*	- Distribution of parasites among case and control groups of children<br>- Relationship between child growth retardation/psychomotor development	- The stool specimens were examined for the presence of parasites, helminth eggs, and larvae and protozoan	

			terms of intestinal parasites, physical growth, and cognitive function.<br>• Controls were from the same community as cases but without the outcome (impaired development and cognition).		complaints and with SDs above −1.99: N=50	*Blastocystis* spp.	delay and intestinal parasitic infection	cysts using direct wet mount, native-Lugol<br>- (saline and Lugol's iodine solution), modified formalin-ethyl acetate sedimentation, and acid-fast stained preparations. The cellophane tapes were examined for the presence of *Taenia saginata* and *Enterobius vermicularis*.<br><br>- Ankara Child Development Screening Inventory (AGTE)	
5	Gari *et al.* (2018)	cohorts	**Inclusion:**<br>- Children in Rift Valley	6-59 months	1st approach: non-stunted N= 2330, non-	Plasmodium parasite	Prevalence of stunting and wasting for the 1st approach	Anthropometry survey; WHZ, HAZ, WAZ	Not reportin g

			area of Ethiopia<br>- 2 cohort: **1st approach**; outcome undernutrition-exposure malaria and **2nd approach**; outcome malaria – exposure undernutrition		wasted N= 4202<br><br>2nd approach; N= 4468		Prevalence of malaria for the 2nd approach	RDT for malaria based on lateral flow immune-chromatography	intestinal parasite
6	Gutiérrez-Jiménez, *et al.* (2019)	Cross section	**Inclusion:**<br>- Children from rural and urban area in mexico<br>-	Under 5	178 children, 84 from Oxchuc-**rural**, and 94 from Chiapa de Corzo - **urban**	*Ascaris lumbricoides*<br>*Entamoeba histolytica/Entamoeba dispar*<br>*Giardia intestinalis*	- Prevalence of intestinal parasites<br><br>- Nutritional assessment	- Identification at the species<br>- level was performed using the API® 20E identification<br>- system<br><br>- WAZ, HAZ, and WHZ	
7	Gyorkos, *et al.* (2011)	Cross sectional	**Inclusion:**<br>- **Children living in Belen-**	7–9 and 12–14 months	Participant: 370 children, only 349 had	Ascaris, Trichuris, hookworm	Prevalence patterns of helminth infection in early childhood	Kato-katz	

			**Peru, highly endemic area**		parasitological results		Association between helminth infection and socio-demographic characteristics<br>Effects of infection on stunting and anemia	Questionnaire to obtain socio-demographic characteristics<br><br>HAZ and blood sample	
8	Haratipour, *et al.* (2016)	cross-sectional	**Inclusion:**<br>- **Children without chronic diseases and no specific drug consumption**<br>- **Pre-school in east area of Iran**	4-6 year old	N= 1850<br>955 (51.6%) boys and<br>895 (48.4%) girls	*Giardia lambia, Hymenolepis nana, Oxyuris, Entamoeba coli,* etc	- Prevalence of intestinal parasites<br><br>Prevalence of malnutrition	Formalin-ether concentration and direct (wet mount Ringer's solution and merthiolate iodine-formalin) techniques. The adhesive cellophane tape (Graham) method was used to diagnose oxyuriasis<br><br>WFH, HFA,	
9	Hegazy, *et al.* (2014)	cross sectional	**Inclusion:**<br>**Children in Damanhur City, Egypt**	2-6 years	N= 500<br>Non-infected= 241<br>Infected= 259	**Cysts** of *Entamoeba histolytica* and *Giardia lamblia*<br>**Ova** of *Ascaris lumbricoides, Enterobius vermicularis,*	Prevalence of intestinal parasitic infection<br>Infection effect on children nutritional status	detailed questionnaire, complete clinical and anthropometric assessment<br>stool analysis and blood picture	

						Ancylostoma duodenale and *Hymenolepis nana*			
10	Lima, *et al.* (1992)	cohort	**Inclusion:** **Young children** presenting to the outpatient clinic at Hospital das Clinicas **in Fortaleza with diarrhea** that had persisted **longer than 14 days**	1 to 29 months	N=30	*Cryptosporidium*, enteroadherent *E. coli*	Nutritional status<br>Prevalence pf intestinal parasite and pathogen	WAZ<br>Cryptosporidium : monoclonal IFA and modified acid fast staining from stool. Giardia: microscopically detected by direct smear.<br>Rotavirus antigen: detected from fecal specimen by ELISA<br>E.coli tested for r hydrophobicity	
11	Lima, *et al.* (2000)	Cohort and case-control	**Inclusion:**<br>**Children in northeastern brazil**<br>**Children that have diarrhea**	0–3 years	189 in cohort, 52 experienced at least 1 episode of PD/ From the 52, 3 could not be matched, and 11 had been designated as controls before onset	*Cryptosporidium, Giardia lamblia, Microsporidium, Ascaris, Trichuris, Entamoeba histolytica*	Nutritional assessment<br>Etiologic studies of diarrheal illness episodes	WAZ, HAZ, and WHZ<br>Microscopy for parasites and leukocytes by use of iodine-stained and methylene blue–stained wet-mount preparations.<br>Modified acid-fast stain for	

					of PD. This gave a total of **38 case-control pairs** for the analysis			*Cryptosporidium* and *Isospora belli*; modified trichrome stain for microsporidium	
12	Lin, *et al.* (2013)		**Inclusion:** Bangladeshi children that lives across **rural Bangladesh** living in different levels of household environmental cleanliness defined by objective indicators of water quality and sanitary and hand-washing infrastructure	≤48 months	N=119	*Ascaris, Trichuris, Giardia*	• Parasite assays<br>• Intestinal permeability assay<br>• Immunological assays<br>• Household water, sanitation, and hygiene conditions<br>• Anthropometrics	• ELISA and direct microscopy<br>• HPLC + PAD<br>• ELISA<br>• Field workers observational spot checks<br>• HAZ, WAZ, WHZ, and HCZ*<br><br>*head circumference-for-age z score	
13	Lunn (1991)		**Inclusion:** Children in developing country, Gambia	2-15			Intestinal integrity<br>Anthropometrics	Non-invasive lactose-mannitol permeability test<br>Height and weight	No info regarding total number of

			Growth flatering after diarrhoea						children and parasite type
14	Moffat (2003)	cross-sectional and longitudinal	**Inclusion:**<br>• Children under five years of age living in periurban Kathmandu, Nepal<br>• The children's parents are all carpet-making workers who live in an environment with crowded living conditions, poor sanitation, and contaminated water.	Under 5 years old	N= 71<br>No parasite found= 41<br>Parasite found= 30	Protozoa: Giardia lamblia, Entamoeba histolyca, etc<br>Nematoda: *Ascaris, Trichuris,* etc	Anthropometrics<br>Morbidity	HAZ, WAZ, and WHZ<br>Maternal reports, and a subsample of children's stools were examined for gastrointestinal parasites	
15	Moore *et al.* (2001)	Prospective cohort	**Inclusion:**<br>Children born in Northeast Brazilian shantytown Goncalves Dias from August 1989 to December 1998	0-2 years old	N=119	*Ascaris* and *Trichuris*	Illness surveillance<br>Nutritional assessment<br>Helminths evaluation	Field workers observation – visited each study home to record diarrhoea<br>HAZ<br>Wet mount, stained with	

			Completed surveillance data and reach the age of 2 years old by December 1998					iodine for microscopy	
16	Moore *et al.* (2010)	cohort	**Inclusion:**<br>Children from shantytown in Brazil, Goncalves Dias<br>The study followed from birth for **10 years** (August 1989 to March 2000)	Less than 5 years old	N= 414	*Cryptosporidium* sp.<br>*Giardia lamblia*<br>*Ascaris* sp,<br>*Trichuris* sp.	Diarrhea/illness surveillance<br>Enteric pathogens<br>Anthropometry/Nutritional assessment	Direct observation from visits<br>Microscopy, ELISA, and various other methods<br>WAZ, HAZ, and WHZ	
17	Vonaesch *et al.* (2017)	Cross-sectional	**Inclusion:**<br>(1) aged between 0–59 months;<br>(2) no history of diarrhoea or antibiotics in the 7 days prior to inclusion;<br>(3) in good general health;<br>(4) recruited in the community, Bangui area<br>(5) written consent by the legal representative to	Less than 5 years	N= 422<br>4 data missing to calculate HAZ<br>4 data outliners<br>Total final data included in the study= 414<br>Non-stunted= 266<br>Stunted= 148	*Cryptosporidium parvum/hominis*<br>*Giardia intestinalis*	Demographic,<br>Socio-economic<br>Anthropometric data<br>Asymptomatic enteropathogen carriage	Survey<br>HAZ, WAZ, zBMI, and WHZ<br>Classical microbiological assays	

			participate in the study						
18	Yoseph (2020)	Cross-sectional	**Inclusion:** Children with caregivers who resided in the Woreda for 6 months Healthy and didn't receive any treatment for 1 month for any diseases	6 to 59 months old	N= 622	*Giardia lamblia* *Entamoeba histoltica* *Ascaris lumbricoides* *Trichiuris trichiuria* Hookworm *Taenia* species *Strongliod stercoralis*	Dietary diversity score and nutritional status of children Prevalence of under-nutrition Prevalence of intestinal parasitic infections	HFA, WFA, and WFH Microscopically for the existence of eggs, trophozoites or cysts by using the direct wet mount, Kato Katz and staining technique	

Table S2. Quality Assessment of the Study based on Joanna Briggs Institute Cross Sectional Study Appraisal Checklist

**Study**	1	2	3	4	5	6	7	8	**Overall Appraisal**	**Reason for Exclusion**
Aiemjoy et al. (2017)	Yes	Yes	Yes	Yes	Yes	Yes	Yes	Yes	Include	
Ajjampur et al. (2011) India	Yes	Yes	Yes	Yes	Yes, not related	Yes, not related	Yes	Yes	Exclude	History of infection not related to main outcome: physical growth parameters
Caron, *et al.* (2018)	Yes	Yes	Yes	Yes	Yes	Yes	Yes	Yes	Include	
Gutiérrez-Jiménez, *et al.* (2019)	Yes	Yes	Yes	Yes	No	No	Yes	Yes	Include	
Gyorkos, *et al.* (2011)	Yes	Yes	Yes	Yes	Yes	Yes	Yes	Yes	Include	
Haratipour, *et al.* (2016)	Yes	Yes	Yes	Yes	No	No	Yes	Yes	Include	
Hegazy, *et al.* (2014)	Yes	Yes	Yes	Yes	No	No	Yes	Yes	Include	
Lima, *et al.* (1992)	Yes	Yes	Yes	Yes	No	No	Yes	Statistical method not described	Exclude	The direct correlation measured was between prolong diarrhoea and nutritional status
Lima, *et al.* (2000)	Yes	Yes	Yes	Yes	No	No	Yes	Yes	Exclude	The direct correlation measured was between prolong

										diarrhoea and nutritional status
Lin, *et al.* (2013)	Yes	Yes	Yes	Yes	Yes	Yes	Yes	Yes	Exclude	The direct correlation measured was between contaminated environment and nutritional status
Moffat (2003)	Yes	Yes	Yes	Yes	Yes	Yes	Yes	Yes	Include	
Moore *et al.* (2001)	Yes	Yes	Yes	Yes	Yes	Yes	Yes	Statistical method not described<br><br>p-values not shown	Include	The direct correlation measured was between acute, prolonged, and persistent diarrhoea and nutritional status
Moore *et al.* (2010)	Yes	Yes	Yes	Yes	Yes	Yes	Yes	Yes	Exclude	The direct correlation measured was between acute, prolonged, and persistent diarrhoea and nutritional status
Vonaesch *et al.* (2017)	Yes	Yes	Yes	Yes	Yes	Yes	Yes	Yes	Include	
Yoseph (2020)	Yes	Yes	Yes	Yes	Yes	Yes	Yes	Yes	Include	

1. Were the criteria for inclusion in the sample clearly defined?
2. Were the study subjects and the setting described in detail?

3. Was the exposure measured in a valid and reliable way?
4. Were objective, standard criteria used for measurement of the condition?
5. Were confounding factors identified?
6. Were strategies to deal with confounding factors stated?
7. Were the outcomes measured in a valid and reliable way?
8. Was appropriate statistical analysis used?

Table S3. Quality Assessment of the Study based on Joanna Briggs Institute Case Control Study Appraisal Checklist

**Study**	1	2	3	4	5	6	7	8	9	10	**Overall Appraisal**	**Reason for Exclusion**
Doni *et al.* (2015)	Yes	Yes	Yes	Yes	Yes	No	No	Yes	Yes	Yes	Include	

1. Were the groups comparable other than the presence of disease in cases or the absence of disease in controls?
2. Were cases and controls matched appropriately?
3. Were the same criteria used for identification of cases and controls?
4. Was exposure measured in a standard, valid, and reliable way?
5. Was exposure measured in the same way for cases and controls?
6. Were confounding factors identified?
7. Were strategies to deal with confounding factors stated?
8. Were outcomes assessed in a standard, valid, and reliable way for cases and controls?
9. Was the exposure period of interest long enough to be meaningful?
10. Was appropriate statistical analysis used?
